# Apelin-13 Pretreatment Promotes the Cardioprotective Effect of Mesenchymal Stem Cells against Myocardial Infarction by Improving Their Survival

**DOI:** 10.1155/2022/3742678

**Published:** 2022-03-21

**Authors:** Guona Chen, Xiaoting Liang, Qian Han, Cong Mai, Linli Shi, Zhuang Shao, Yimei Hong, Fang Lin, Mimi Li, Bei Hu, Xin Li, Yuelin Zhang

**Affiliations:** ^1^School of Medicine, South China University of Technology, Guangzhou, China; ^2^Department of Emergency Medicine, Guangdong Provincial People's Hospital, Guangdong Academy of Medical Sciences, Guangzhou, China; ^3^Institute for Regenerative Medicine, Shanghai East Hospital, School of Life Sciences and Technology, Tongji University, Shanghai, China; ^4^Department of Respiratory Medicine, The First Affiliated Hospital of Guangzhou Medical University, Guangzhou Institute of Respiratory Health, State Key Laboratory of Respiratory Disease, Guangzhou, China; ^5^Research Center for Translational Medicine, Shanghai East Hospital, School of Medicine, Tongji University, Shanghai, China

## Abstract

Although mesenchymal stem cell- (MSC-) based therapy has shown promising results for myocardial infarction (MI), low cell survival heavily limits its beneficial effects. Apelin plays an essential regulatory role in cell proliferation. This study was aimed at determining whether Apelin-13 pretreatment could improve the survival of MSCs in the ischemic heart and enhance their cardioprotective efficacy against MI. MSCs were pretreated with or without Apelin-13 for 24 hours and then exposed to serum deprivation and hypoxia (SD/H) for 48 hours. The mitochondrial morphology of MSCs was assessed by MitoTracker staining. The apoptosis of MSCs was determined by TUNEL staining. The level of mitochondrial reactive oxygen species (ROS) of MSCs was detected by Mito-Sox staining. MSCs and Apelin-13-pretreated MSCs were transplanted into the peri-infarct region in a mouse MI model. Apelin-13 pretreatment protected MSCs against SD/H-induced mitochondrial fragmentation and apoptosis. Apelin-13 pretreatment reduced ROS generation induced by SD/H in MSCs. Furthermore, Apelin-13 pretreatment enhanced the angiogenesis of MSCs under SD/H conditions. Mechanistically, Apelin-13 pretreatment inhibited SD/H-induced MSC apoptosis by downregulating mitochondrial fission via activation of the ERK pathway, and these effects were partially abrogated by ERK inhibitor U0126. Apelin-13 pretreatment promoted the survival of MSCs in the ischemic heart. Moreover, transplantation with Apelin-13-pretreated MSCs improved heart function and increased angiogenesis accompanied by decreased fibrosis compared with MSC transplantation at 28 days following MI. These findings reveal that pretreatment with Apelin-13 improves MSCs survival and enhances their therapeutic efficacy for MI. Our study provides a novel approach to improve MSC-based therapy for cardiovascular disease.

## 1. Introduction

Myocardial infarction (MI) is a leading cause of mortality and morbidity worldwide. Despite advanced medical and surgical interventions for MI, the only well-established therapy is heart transplantation although this is heavily limited by a shortage of donor hearts, high cost, and inevitable immune rejection after transplantation [1]. Over the past decades, accumulating preclinical studies and clinical trials have proven that stem cell-based therapy is a promising strategy for treating MI [2–4]. Among all the types of stem cells used for MI treatment, mesenchymal stem cells (MSCs) have attracted considerable attention due to their excellent biological properties including easy isolation, low immunogenicity, and multiple differentiation potential [5, 6]. Nonetheless, the ischemic microenvironment in heart tissue seriously restricts MSC survival posttransplantation, thereby limiting their cardioprotective effects in MI [7–9]. There is an urgent need to explore new approaches to improve MSC survival after transplantation in the ischemic heart. Indeed, pharmacologic pretreatment has been proposed as a novel strategy [10–12].

Apelin, an endogenous polypeptide ligand for the orphaned G protein-coupled receptor APJ, is widely expressed in multiple organs, including the heart [13]. It has several isoforms of which Apelin-13 is the most active one, binding to the APJ receptor. It is well documented that Apelin plays an essential role in regulating the biological properties of MSCs [14, 15]. In a mouse model of hindlimb ischemia, Apelin treatment increased the viability of adipose-derived MSCs by promoting autophagy during hypoxia via activation of the Akt signaling pathway, leading to a better therapeutic efficacy [16]. Furthermore, overexpression of Apelin rejuvenated the aged-MSCs and enhanced their cardioprotective effects in MI [17]. Nonetheless, whether pretreatment of Apelin-13 can enhance the survival of MSCs in the ischemic heart and thereby improve cardiac repair and the underlying mechanisms remains unclear.

The mitochondrion is a dynamic organelle regulated by fission and fusion. There is an increasing recognition that mitochondrial fission-induced reactive oxygen species (ROS) contribute to cell apoptosis, whereas inhibition of mitochondrial fission protects against apoptosis under stressed conditions [18]. Our previous study showed that serum deprivation and hypoxia (SD/H) induced apoptosis of MSCs by upregulation of mitochondrial fission via regulation of HO-1, and these effects were partially abrogated by hemin pretreatment [19]. Interestingly, there is an increasing evidence that Apelin plays a critical role in regulating mitochondrial function and thereby exerts beneficial effects in various diseases [20–22]. Nonetheless, whether Apelin-13 pretreatment protects against SD/H-induced mitochondrial fission in MSCs remains unknown. This study was aimed at investigating whether Apelin-13 pretreatment could improve the survival of MSCs in the ischemic heart and enhance their cardioprotective efficacy. We showed that pretreatment with Apelin-13 improved the survival of MSCs by inhibiting mitochondrial fission via regulation of the ERK signaling pathway under SD/H challenge and enhanced their cardioprotective efficacy in MI. Our study provides a new therapeutic strategy to improve MSC-based therapy for MI.

## 2. Materials and Methods

### 2.1. MSC Culture and Apelin-13 Pretreatment

MSCs were cultured as previously described [17]. Briefly, human bone marrow was obtained from volunteer donors with informed consent in the current study. This study was approved by the research ethics board of Shanghai East Hospital (No. 2016-050). MSCs were isolated and regularly cultured in Dulbecco's minimum essential medium- (DMEM-) high glucose (11965084, Gibco) supplemented with 10% fetal bovine serum (FBS; 16000, Life Technologies), 5 ng/mL endothelial growth factor (AF-100-15, PeproTech), and 5 ng/mL basic fibroblast growth factor (100-18B, PeproTech) as previously reported [17]. MSCs at passage 3-4 were used in this study. For Apelin-13 (HY-P1944A, MCE) pretreatment, MSCs were cultured in fresh complete medium supplemented with different concentrations of Apelin-13 (0.01, 0.05, 0.1, 0.5, and 1 nM) under normoxia (95% air and 5% CO_2_) for 24 hours and then exposed to serum deprivation and hypoxia (SD/H) for 48 hours. The SD/H challenge was generated as previously described [19]. Briefly, MSCs at 70%-80% confluence were maintained in medium without FBS and cultured under hypoxia (1% oxygen, 5% carbon dioxide, and 94% nitrogen) for 48 hours.

### 2.2. Cell-Counting Kit-8 (CCK-8) Assay

The impact of Apelin-13 on MSC viability under normoxia or SD/H challenge was evaluated by CCK-8 assay (C0038, Beyotime Biotechnology) according to the manufacturer's protocol. Briefly, 4 × 10^3^ MSCs were plated on 96-well plates and cultured with or without different concentrations of Apelin-13 for 24 hours in fresh complete medium under normoxia or followed by SD/H challenge for 48 hours. Subsequently, MSCs were incubated with CCK-8 solution at 37°C in a dark place for 2 hours. Finally, the optical density (OD) values were measured at 450 nm with a microplate reader (Biotek).

### 2.3. MSC and Spleenocyte Coculture

MSCs and Apelin-13-MSCs were seeded into a 96-well plate for 4 hours to allow adherence. Mouse (C57/B6J) spleenocytes were then added to the MSC pre-seeded plates. Spleenocytes cultured with normal medium were applied as the control group. After 72 hours of coculture, the suspended spleenocytes were aspirated and seeded to a new plate. Spleenocytes proliferation was quantified using a bromodeoxyuridine (BrdU) kit according to the manufacturer's instructions (Roche Applied Science, 11647229001).

### 2.4. Mito-Sox Staining

The mitochondrial reactive oxygen species (ROS) generation of MSCs with different treatments was measured by Mito-Sox staining according to the protocol. Briefly, MSCs were cultured in 24-well plates with glass coverslips and different treatments were administered. After washing with phosphate buffered saline (PBS), MSCs were cultured with 5 *μ*M Mito-Sox (M36008, Invitrogen) at 37°C in the dark for 15 minutes. Next, using a fluorescent microscope, the sample was randomly captured from five different view fields of each group. Finally, the fluorescence intensity was analyzed using ImageJ software.

### 2.5. MitoTracker Staining

The mitochondrial morphology of MSCs was assessed by MitoTracker Green FM (M7514, Invitrogen). Briefly, MSCs were cultured in 24-well plates with glass coverslips and different treatments applied. Next, MSCs were cultured with DMEM containing 20 nM MitoTracker Green FM for 15 minutes. After washing with PBS three times, MSCs were randomly captured under a confocal microscope. Cell nucleus were identified as a vacuole surrounded by the stained mitochondria. Number of mitochondria and mean mitochondria area were calculated using methods as previously described [23]. Six fields were randomly chosen, and at least 300 cells per treatment group were counted.

### 2.6. Annexin V/PI Staining

The apoptosis of MSCs with different treatments was evaluated using the Annexin V/PI (propidium iodide) apoptosis detection kit (AD10, DOJINDO). In brief, MSCs were seeded in 6-well plates (1.5 × 10^5^/well) and different treatments were applied, with three replicates in each group. After the intervention, the cells were digested and stained with 5 *μ*L Annexin V and 5 *μ*L PI for 15 minutes at room temperature and protected from light. The apoptotic rate was analyzed by flow cytometry (Beckman cytoFLEX).

### 2.7. HUVEC Tube Formation Analysis

The conditioned medium (CdM) of MSCs was prepared as previously described [24]. Briefly, MSCs were seeded in 6-well plates and cultured until they reached 70-80% confluence, then treated with PBS or Apelin-13 or Apelin-13+U0126 (10 *μ*M; HY-12031A, MCE) for 24 hours. Next, the medium was replaced with 2 mL serum-free medium per well and cultured for a further 48 hours under SD/H. Subsequently, the CdM was harvested, centrifuged, and stored at −80°C until use. HUVECs (human umbilical vein endothelial cells, 30 000 cells/well) were seeded in a 96-well plate coated with growth-factor-reduced matrigel (356230, BD Biosciences). Next, HUVECs were treated with MSC-CdM, Apelin-13-MSC-CdM, or Apelin-13+U0126-MSC-CdM for 6 hours. Finally, the capillary-like tube formation was photographed (magnification ×100). The endothelial tube length of different groups was analyzed using ImageJ software.

### 2.8. Western Blotting

MSCs were cultured under normoxia or SD/H conditions as described above. In indicated groups, 10 *μ*M U0126 (HY-12031A, MCE) or 10 *μ*M ML221 (HY-103254, MCE) was added together with Apelin-13. The protein of MSCs with different treatments was extracted using RIPA buffer (9806, CST), and the concentration of each sample was determined using a bicinchoninic acid assay kit (231227, Thermo). A total of 30 *μ*g protein from each sample was separated on SDS-PAGE gel and transferred to PVDF membranes. After washing with Tris-buffered saline containing 0.1% Tween-20 (TBST) three times, the membranes were blocked by TBST with 5% fat-free milk and then incubated overnight at 4°C with the following primary antibodies: anti-p-ERK (9101, CST), anti-t-ERK (4695, CST), anti-Drp1 (PA5-20176; Invitrogen), anti-p-Drp1 ser616 (PA5-64821, Invitrogen), anti-Mfn1 (ab57602, Abcam), anti-Mfn2 (ab124773, Abcam), anti-APJ (20341-1-AP, Proteintech), and anti-GAPDH (2118, CST). Subsequently, the membranes were washed with TBST three times and incubated with horseradish peroxide-conjugated secondary antibodies at room temperature for 1 hour. Finally, the membranes were exposed by enhanced chemiluminescence (ECL plus) (Amersham) in a dark room.

### 2.9. MI Model and MSC Transplantation

All animal experiments were approved by the Committee on the Use of Live Animals in Teaching and Research of the Tongji University for Laboratory Animal Medicine (No. TJBB00120102). The animal model of MI was induced in adult C57/B6J mice (6–8 weeks) by ligation of the left anterior descending coronary artery (LAD) with an 8-0 silk suture as previously described [25, 26]. After LAD ligation for 30 minutes, mice were randomly allocated to receive one of the following treatments: (1) PBS (MI group, (*n* = 12); (2) 3 × 10^5^ MSCs (MSC group, *n* = 12); or (3) 3 × 10^5^ Aplein-13-pretreated MSCs (Apelin-13-MSC group, *n* = 12). All MSCs were suspended in 40 *μ*L PBS and injected at four sites of the surrounding border of the infarcted area. Mice that underwent thoracotomy without LAD ligation were served as the sham group.

### 2.10. Echocardiographic Measurement

Heart function in each mouse was determined at 4 weeks following MI by transthoracic echocardiography (Ultramark 9; Soma Technology). Left ventricle ejection fraction (LVEF) and fraction shorting (LVFS) were calculated.

### 2.11. Masson's Staining

After measuring heart function at 28 days post-MI, all mice were sacrificed and heart tissue was collected, embedded, and sectioned into 5 *μ*m slices. Next, Masson's staining was performed according to the manufacturer's protocol (HT15, Sigma). The percentage of fibrosis was calculated as the ratio of fibrosis area to total LV area × 100%.

### 2.12. Immunofluorescence Staining

The heart sections from different groups were hydrated and the antigens were retrieved. Next, the sections were permeabilized with 0.1% Triton X100 for 30 minutes and blocked with 5% bovine serum albumin for 30 minutes. Subsequently, heart sections were incubated overnight at 4°C with the following primary antibodies: anti-human mitochondria (1 : 100; ab92824, Abcam), anti-CD31 (1 : 100; 77699, CST), and anti-*α*-smooth muscle actin (*α*-SMA) (1 : 100; ab5694, Abcam). After washing with TBST three times, the slides were incubated with fluorescent secondary antibodies (thermo fisher) for 30 minutes at room temperature. Finally, human mitochondria density, capillary or arteriole density was evaluated using the average number of human mitochondria positive cells, CD31- or *α*-SMA-positive blood vessels per field (magnification ×100).

### 2.13. Polymerase Chain Reaction (PCR)

The expression levels of APJ and the proangiogenesis cytokines including v*ascular endothelial growth factor A* (*VEGFA*), *fibroblast growth factor 2* (*FGF2*), and *platelet derived growth factor subunit B* (*PDGFB*) were determined by quantitative real-time polymerase chain reaction (qRT-PCR). Briefly, total RNA was extracted from with TRIzol reagent (Takara, 2270A). cDNA was synthesized from 500 ng of total RNA with a PrimeScript RT Reagent Kit (RR037A, Takara)). Then, qRT-PCR analysis was performed with SYBR Green Master Mix (Q111-02, Vazyme) in an ABI QuantStudio 6 Flex System. The relative standard curve method (2△△^ct^) was used to determine the relative mRNA expression, with glyceraldehyde 3-phosphate dehydrogenase (GAPDH) as the reference gene. The primer sequences were listed as follows: *APJ*-F: 5′-ACTTCCGCAAGGAACGCATCGA-3′, *APJ*-R: 5′-ACAGCGTCTTCACCAGGTGGTA-3′. *VEGFA*-F: 5′-AGGGCAGAATCATCACGAAGT-3′, *VEGFA*-R: 5′-AGGGTCTCGATTGGATGGCA-3′; *FGF2*-F: 5′-AGAAGAGCGACCCTCACATCA-3′, *FGF2*-R: 5′-CGGTTAGCACACACTCCTTTG-3′; *PDGFB*-F: 5′-CTCGATCCGCTCCTTTGATGA-3′, *PDGFB*-R: 5′-CGTTGGTGCGGTCTATGAG-3′； *GAPDH*-F: 5′-GGAGCGAGATCCCTCCAAAAT-3′, *GAPDH*-R: 5′-GGCTGTTGTCATACTTCTCATGG-3′. Genomic PCR for human Alu-sx repeat sequences in heart tissue from different groups was performed as previously described [19]. The primer of human Alu-sx was F: 5′- GGCGCGGTGGCTCACG-3′, R: 5′-TTTTTTGAGACGGAGTCTCGCTC-3. The amplified products were examined by electrophoresis in 1.5% agarose gel supplemented with ethidium bromide.

### 2.14. Statistical Analysis

All data are shown as mean ± standard deviation (SD). Statistical analyses were performed using Prism 5.04 software (GraphPad Software for Windows, San Diego, CA, USA). Unpaired Student's *t*-test was used for comparisons between two groups. A one-way ANOVA followed by the Bonferroni test was used for comparisons between more than two groups. A value of *p* < 0.05 was considered statistically significant.

## 3. Results

### 3.1. Apelin-13 Pretreatment Inhibited SD/H-Induced Mitochondrial Fragmentation and Apoptosis of MSCs

**Figure 1:**
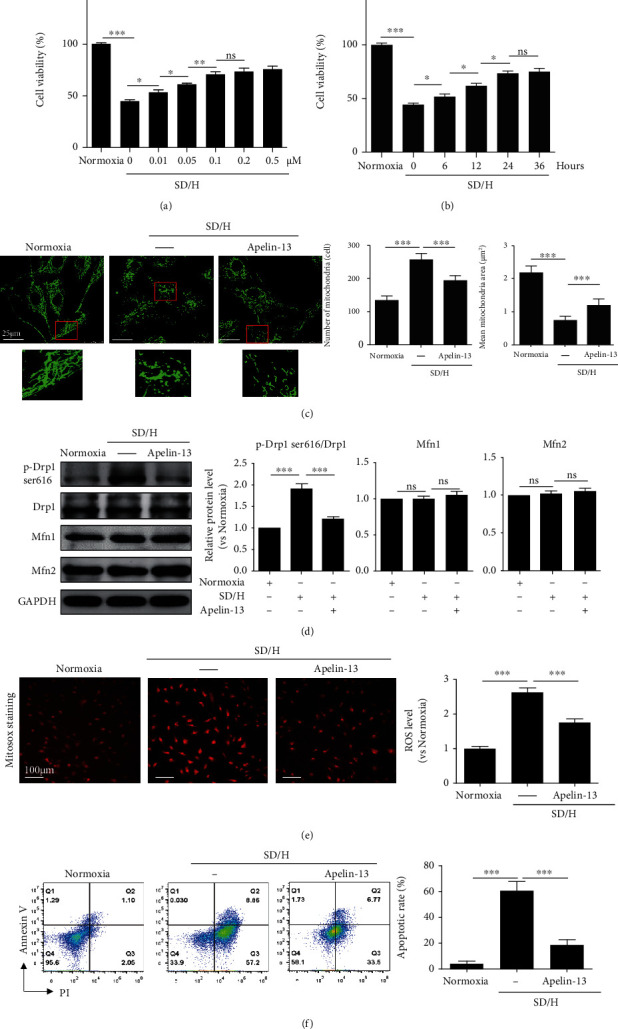
Apelin-13 pretreatment inhibits SD/H-induced mitochondrial fragmentation and apoptosis of MSCs. (a) CCK-8 assay showed the cell viability of MSCs with or without Apelin-13 (0.01, 0.05, 0.1, 0.5, and 1 nM) pretreatment for 24 hours under normoxic or SD/H conditions. (b) CCK-8 assay showed the cell viability of MSCs with or without 0.1 nM Apelin-13 pretreatment for 6, 12, 24, or 36 hours under normoxic or SD/H conditions. (c) Representative images of MitoTracker staining and quantitative analysis of the mitochondrial number and mean mitochondrial area in MSCs and Apelin-13-pretreated MSCs under normoxic or SD/H conditions. (d) Western blotting and quantitative analysis of the protein level of Mfn1/2 and p-Drp1 ser616 in MSCs and Apelin-13-pretreated MSCs under normoxia or SD/H challenge. (e) Representative images of Mito-Sox staining and quantitative analysis of ROS generation in MSCs and Apelin-13-pretreated MSCs under normoxic or SD/H conditions. (f) Representative images of Annexin V/PI staining and quantitative analysis of the apoptotic rate in MSCs and Apelin-13-pretreated MSCs under normoxic or SD/H conditions. *n* = 3 biological replicates for each group. Data are expressed as mean ± SD. ^∗^p < 0.05, ^∗∗^p < 0.01, ^∗∗∗^p < 0.001. ns, not significant.

We determined the concentration-response curve for Apelin-13 in MSCs under normoxia by CCK8 assay. Under normoxic condition, MSCs were treated with different concentrations of Apelin-13 (0.01, 0.05, 0.1, 0.5, and 1 nM) and cultured for 48 hours. As shown in supplementary Figure 1, Apelin-13 at concentration of 0.01, 0.05, 0.1, 0.5, and 1 nM did not affect the proliferation curve of MSCs during the 48 hours observation period under normoxia. To determine whether Apelin-13 pretreatment could protect MSCs under conditions of SD/H, we pretreated MSCs with different concentrations of Apelin-13 (0.01, 0.05, 0.1, 0.5, and 1 nM) for 24 hours and then exposed them to SD/H for 48 hours. The CCK-8 assay showed that Apelin-13 pretreatment had a dose-dependent protective effect on MSCs under SD/H challenge, with the highest cell viability reached at a concentration of 0.1 nM (Figure 1(a)). Pretreatment with 0.1 nM Aplein-13 for 24 hours elicited the highest cell viability of MSCs under SD/H challenge (Figure 1(b)). Therefore, 24-hour pretreatment with 0.1 nM Apelin-13 was determined as the optimal treatment for MSCs in subsequent studies. We also evaluated the effects of Apelin-13 pretreatment on MSC immunogenicity *in vitro*. In a coculture setting, MSCs and Apelin-13-MSCs did not induce proliferation in mouse spleenocytes, indicating Apelin-13 pretreatment did not alter MSC immunogenicity (supplementary Figure 2). Our previous study documented that SD/H-induced mitochondrial fragmentation mediates MSC apoptosis [19]. Therefore, we determined whether Apelin-13 pretreatment protected MSCs against SD/H via regulating the mitochondrial dynamics. As shown in Figure 1(c), MSCs under normoxic condition showed an elongated rod-like structure of mitochondria, connecting each other to form visible networks. In contrast, MSCs under SD/H conditions appeared smaller, shorter, and less connected mitochondria, accompanied by an increased mitochondrial number and decreased mitochondrial area, suggesting that SD/H induced mitochondrial fragmentation. Apelin-13 pretreatment significantly ameliorated SD/H-induced mitochondrial fragmentation, as evidenced by the restoration of the elongated rod-shaped structure of the mitochondria, decreased mitochondrial number, and increased mitochondrial area (Figure 1(c)). Western blotting showed that the SD/H challenge significantly increased the mitochondrial fission-related protein p-Drp1 ser616, and this effect was partially reversed by Apelin-13 pretreatment (Figure 1(d)). Nonetheless, SD/H challenge had no impact on the mitochondrial fusion-related protein Mfn1/2 (Figure 1(d)). Similarly, Apelin-13 pretreatment inhibited mitochondrial ROS generation induced by SD/H (Figure 1(e)) and markedly reduced SD/H-induced apoptosis (Figure 1(f)). Collectively, these data showed that Apelin-13 pretreatment inhibited the mitochondrial fragmentation and apoptosis of MSCs under the conditions of SD/H.

### 3.2. Apelin-13 Pretreatment Inhibits Mitochondrial Fragmentation of MSCs Induced by SD/H via Regulating the ERK Signaling Pathway

**Figure 2:**
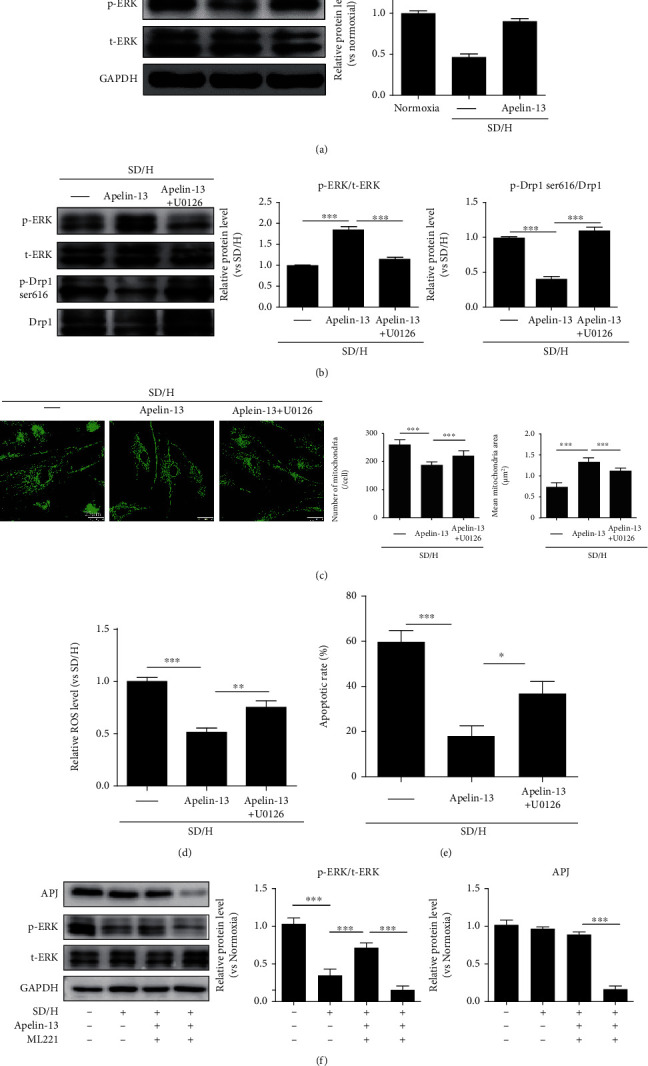
Apelin-13 pretreatment inhibits SD/H-induced mitochondrial fragmentation in MSCs via regulation of the ERK signaling pathway. (a) Western blotting and quantitative analysis for p-ERK and t-ERK protein level in MSCs and Apelin-13-pretreated MSCs under normoxic or SD/H conditions. (b) Western blotting and quantitative analysis for p-ERK, t-ERK, p-Drp1 ser616, and Drp1 protein level in MSCs, Apelin-13-pretreated MSCs, or Apelin-13-pretreated MSCs with U0126 under SD/H condition. (c) Representative images of MitoTracker staining and quantitative analysis of the number of mitochondria and mean mitochondrial area in MSCs, Apelin-13-pretreated MSCs or Apelin-13-pretreated MSCs with U0126 under SD/H condition. (d) Quantitative analysis of ROS generation in MSCs, Apelin-13-pretreated MSCs, or Apelin-13-pretreated MSCs with U0126 under SD/H condition. (e) Quantitative analysis of the apoptotic rate of MSCs, Apelin-13-pretreated MSCs, or Apelin-13-pretreated MSCs with U0126 under SD/H condition. (f) Western blotting and quantitative analysis for APJ, p-ERK, and t-ERK protein level in MSCs, Apelin-13-pretreated MSCs, or Apelin-13-pretreated MSCs with ML221 under normoxic or SD/H conditions. *n* = 3 biological replicates for each group. Data are expressed as mean ± SD. ^∗∗^p < 0.01, ^∗∗∗^p < 0.001.

Next, we explored the molecular mechanisms by which Apelin-13 pretreatment regulated mitochondrial fission in MSCs. The ERK signaling pathway plays an essential role in regulating mitochondrial fission [27, 28]. Western blotting showed that Apelin-13 pretreatment significantly improved the downregulation of p-ERK protein level in MSCs under SD/H challenge, indicating that the protective effects of Apelin-13 may be attributed to regulation of the ERK signaling pathway (Figure 2(a)). To further verify whether the ERK signaling pathway is involved in regulating the protective effects of Apelin-13 on MSCs, we treated the Apelin-13-pretreated MSCs with an ERK inhibitor U0126 and then exposed them to SD/H challenge. As shown in Figure 2(b), elevation of p-ERK expression was greatly reduced in Apelin-13-pretreated MSCs with U0126 compared with Apelin-13-pretreated MSCs under SD/H (Figure 2(b)). Nonetheless, the downregulation of p-Drp1 ser616 expression was greatly increased in Apelin-13-pretreated MSCs with U0126 compared with Apelin-13-pretreated MSCs under SD/H (Figure 2(b)). Apelin-13-pretreated MSCs exhibited greatly reduced mitochondrial fragmentation under SD/H, as evidenced by the decreased number of mitochondria and increased mitochondrial area (Figure 2(c)). Apelin-13 pretreatment reduced mitochondrial ROS level and apoptosis of MSCs under SD/H condition (Figures 2(d) and 2(e)). More importantly, the effects of Apelin-13 on mitochondrial fragmentation, mitochondrial ROS level, and apoptosis were partially reversed by U0126 treatment (Figures 2(c)–2(e)). To confirm the Apelin-13 receptor-mediated activation of ERK signaling, we determined the alteration of ERK signaling in the presence of the Apelin/APJ functional antagonist ML221 under SD/H challenge. We first examined APJ expression in MSCs and showed that MSCs derived from different donors expressed comparable level of APJ (supplementary Figure 3). Apelin-13 pretreatment significantly improved the downregulation of p-ERK protein level in MSCs under SD/H challenge (Figure 2(f)). ML221 effectively suppressed the APJ expression, and more importantly, ML221 inhibited the upregulated p-ERK expression induced by Apelin-13 under SD/H challenge, suggesting Apelin-13/APJ mediated the ERK signaling activation (Figure 2(f)). These results showed that Apelin-13 pretreatment inhibited SD/H-induced mitochondrial fragmentation and apoptosis of MSCs by activating the ERK signaling pathway.

### 3.3. Apelin-13 Pretreatment Improves the Angiogenic Capacity of MSCs

**Figure 3:**
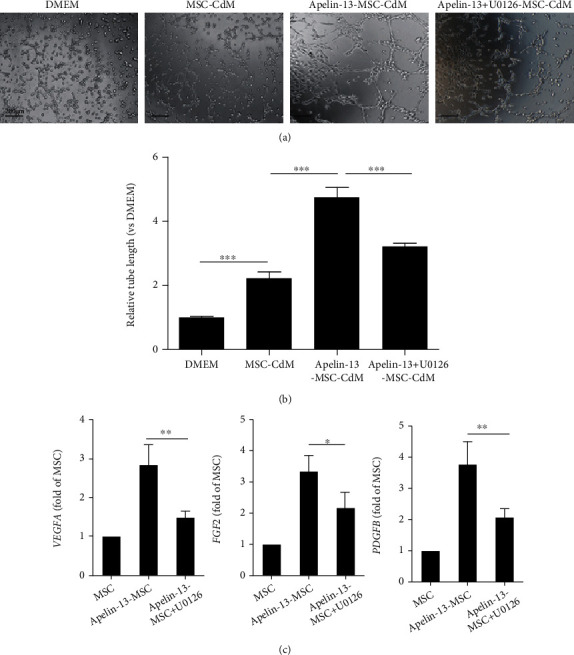
Apelin-13 pretreatment improves the angiogenic capacity of MSCs. (a) Representative light images of HUVEC tube formation assay in MSC-CdM, Apelin-13-MSC-CdM, and Apelin-13+U0126-MSC-CdM treatment. (b) Quantitative analysis of the HUVEC tube length in MSC-CdM, Apelin-13-MSC-CdM, and Apelin-13+U0126-MSC-CdM treatment. (c) Expression of proangiogenesis cytokines determined by qRT-PCR. *n* = 3 biological replicates for each group. Data are expressed as mean ± SD. ^∗^p < 0.05, ^∗∗^p < 0.01, ^∗∗∗^p < 0.001.

Subsequently, to determine whether Apelin-13 pretreatment could improve the angiogenic capacity of MSCs, we collected the CdM from MSCs, Apelin- pretreated MSCs, and Apelin-pretreated MSCs with U0126 treatment and then subjected it to HUVEC tube formation assay. Compared with MSC-CdM, the endothelial tube length was significantly increased in the Apelin-13-MSC-CdM-treated HUVECs, and this effect was largely abrogated by Apelin-13+U0126-MSC-CdM (Figures 3(a) and 3(b)). We measured several proangiogenesis cytokine expression by qRT-PCR. The expression levels of *VEGFA*, *FGF2*, and *PDGFB* were significantly elevated after Apelin-13 pretreatment, and this effect was largely abrogated by U0126 (Figure 3(c)). These results indicated that pretreatment with Apelin-13 enhanced the angiogenic properties of MSCs via activation of the ERK signaling pathway.

### 3.4. Transplantation of Apelin-13-Pretreated MSCs Improved Heart Function following Infarction in Mice

**Figure 4:**
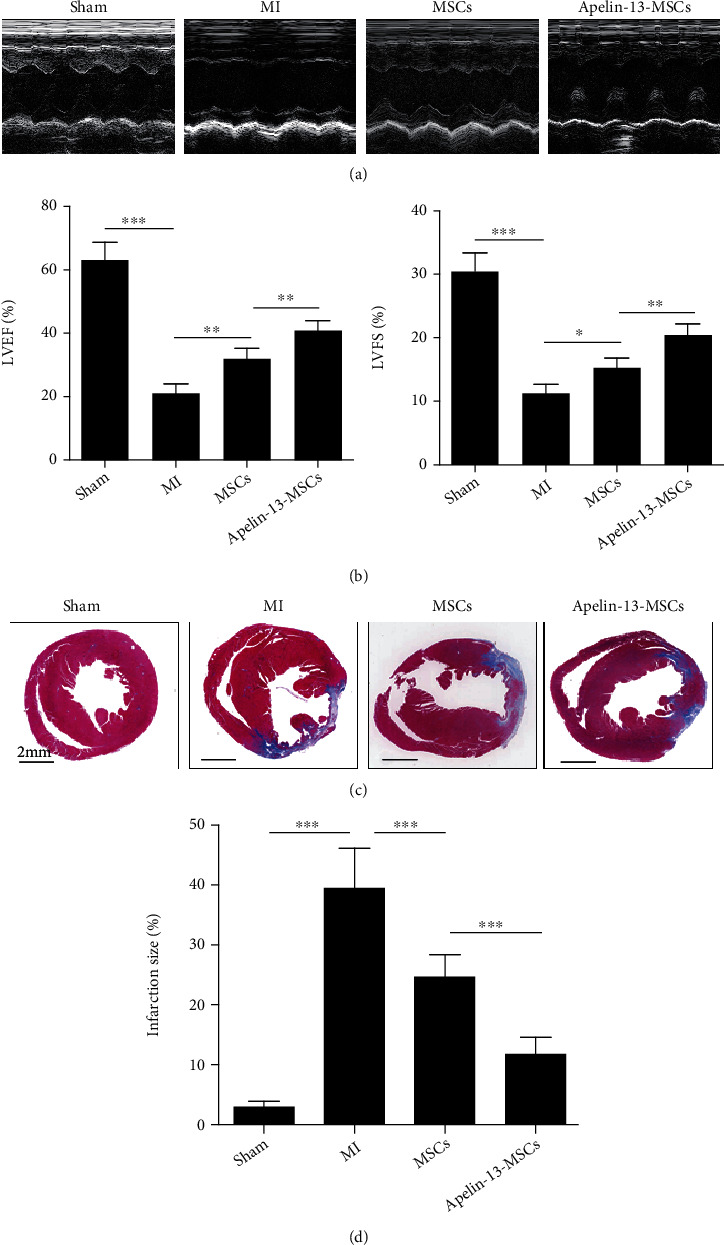
Transplantation of Apelin-13-pretreated MSCs improves heart function following infarction in mice. (a) Representative images of echocardiographic images captured at 4 weeks in mice among the different groups. (b) Quantitative analysis of LVEF and LVFS at 4 weeks in mice from the different groups. (c) Representative images of Masson's trichrome staining of heart sections at 4 weeks in mice among the different groups. (d) Quantitative measurement of heart fibrosis at 4 weeks in mice among the different groups. *n* = 6 mice for each group. Data are expressed as mean ± SD. Scale bar = 2 mm. ^∗^p < 0.05, ^∗∗^p < 0.01, ^∗∗∗^p < 0.001.

At 4 weeks post-MSC transplantation, transthoracic M model echocardiography was performed to evaluate heart function in mice from different groups (Figure 4(a)). Compared with the sham group, left ventricle ejection fraction (LVEF) and fraction shortening (LVFS) were severely reduced in the MI group (Figure 4(b)). Nonetheless, MSC transplantation greatly enhanced LVEF and LVFS in mice following infarction compared with the MI group (Figure 4(b)). Notably, compared with the MSC group, LVEF and LVFS were significantly increased in the Apelin-13-MSC group (Figure 4(b)). The interstitial fibrosis of myocardial tissue sections was evaluated by Masson's trichrome staining at 28 days following MSC transplantation (Figure 4(c)). The interstitial fibrosis of the mouse hearts was greatly increased following MI compared with the sham group, and only a slight decrease was observed following MSC transplantation compared with the MI group (Figure 4(d)). Nonetheless, Apelin-13-pretreated MSC transplantation greatly reduced the fibrotic area compared with the MI group (Figure 4(d)). These results indicate that Apelin-13 pretreatment enhances the cardioprotective effects of MSCs in mice following infarction.

### 3.5. Apelin-13 Pretreatment Enhanced MSC Survival in the Ischemic Heart of Mice

**Figure 5:**
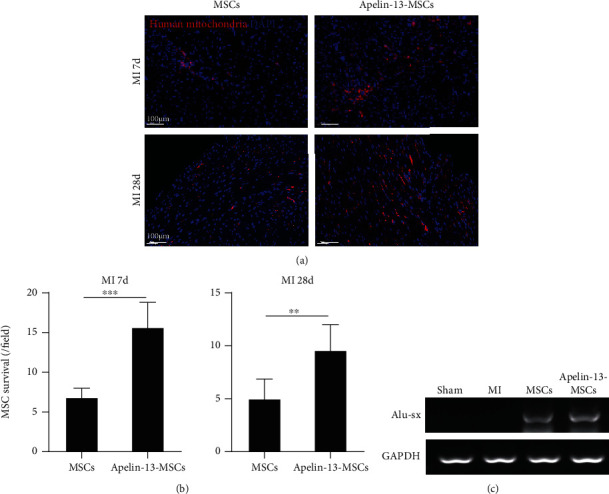
Apelin-13 pretreatment improves MSC survival in mice hearts following infarction. (a) Representative images of human mitochondria staining in the ischemic heart of mice at 7 days and 4 weeks among the different groups. (b) Quantitative analysis of human mitochondria positive cells in ischemic mice hearts at 7 days and 4 weeks among the different groups. (c) Alu-sx expression was evaluated by PCR in the sham, MI, MSCs, or Apelin-13-MSCs group. *n* = 6 mice for each group. Data are expressed as mean ± SD. ^∗∗^p < 0.01, ^∗∗∗^p < 0.001.

The MSC survival in the ischemic heart of mice at 7 days and 4 weeks posttransplantation was examined by anti-human mitochondria staining. As shown in Figure 5(a), positive human mitochondria staining was detected in both MSC and Apelin-13-MSC transplanted mice at 7 days and 4 weeks post-MI (Figure 5(a)). Notably, compared with the MSC group, MSC survival was significantly increased in the Apelin-13-MSC group, indicating that Apelin-13 pretreatment improved the tolerance capacity of MSCs in the presence of ischemia (Figure 5(b)). We also performed PCR of the human repeat sequences Alu-sx to assess MSC survival in ischemic heart tissue at 4 weeks post-MI. As shown in Figure 5(c), Alu-sx was detected in the MSC group and Apelin-13-MSC group, but not in the sham or MI group (Figure 5(c)). Furthermore, the expression of Alu-sx was much higher in the Apelin-13-MSC group than that in the MSC group (Figure 5(c)). Collectively, these data indicate that Apelin-13 pretreatment enhances MSC survival in the ischemic heart of mice.

### 3.6. Transplantation of Apelin-13-Pretreated MSCs Improved Angiogenesis in Mouse Hearts following Infarction

**Figure 6:**
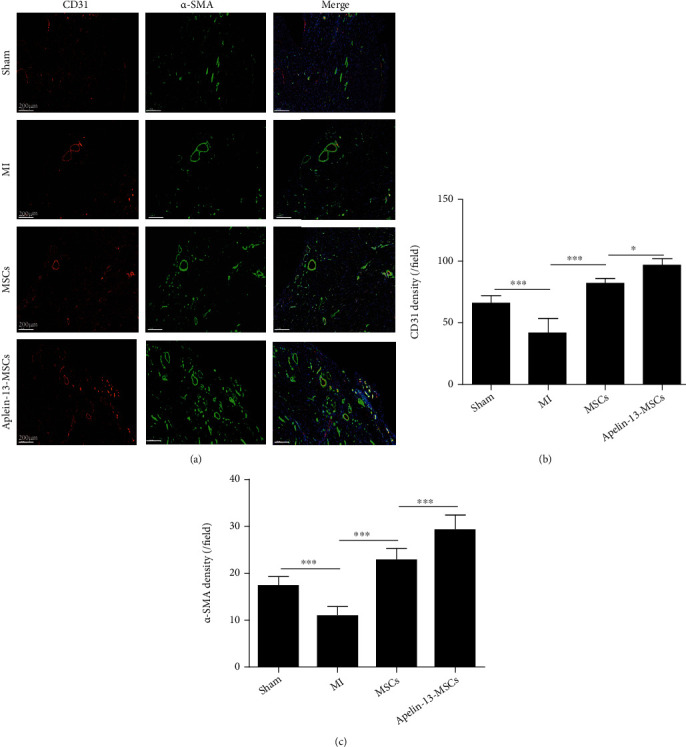
Transplantation of Apelin-13-pretreated MSCs improves angiogenesis in mouse hearts following infarction. (a) Representative images of CD31 staining and *α*-SMA staining in the ischemic heart of mice at 4 weeks among the different groups. (b) Quantitative analysis of the capillary density in the ischemic heart at 4 weeks among the different groups. (c) Quantitative analysis of the arteriole density in the ischemic heart at 4 weeks among the different groups. *n* = 6 mice for each group. Data are expressed as mean ± SD. ^∗^p < 0.05, ^∗∗^p < 0.01, ^∗∗∗^p < 0.001.

Since Apelin-13 pretreatment can enhance the angiogenic capacity of MSCs *in vitro*, we examined the capillary density of the ischemic area post-MSC transplantation using CD31 staining. Compared with the sham group, the capillary density was reduced in the MI group (Figures 6(a) and 6(b)) but slightly increased in the MSC group and greatly increased in the Apelin-13-MSC group (Figures 6(a) and 6(b)). We observed a similar result for arteriole density evidenced by *α*-SMA staining among the different groups (Figures 6(a) and 6(c)). Compared with the sham group, the arteriole density was greatly reduced in the MI group but increased in the two cell transplanted groups (Figures 6(a) and 6(c)). Notably, the arteriole density was much higher in the Apelin-13-MSC group than in the MSC group (Figures 6(a) and 6(c)). Our results showed that transplantation of Apelin-13-pretreated MSCs improved angiogenesis in mouse hearts following infarction.

## 4. Discussion

There are several major findings in the current study. First, Apelin-13 pretreatment significantly reduced the apoptosis of MSCs under SD/H challenge *in vitro* by inhibiting mitochondrial fission via regulation of the ERK signaling pathway. Second, Apelin-13 pretreatment improved the angiogenic capacity of MSCs. Third, Apelin-13 pretreatment greatly improved the survival of transplanted MSCs, thereby enhancing cardiac protection efficacy for MI in mice. Our study revealed that Apelin-13 is a potential cytoprotective factor for MSC-based therapy in MI.

Although MSC transplantation has shown promising results in treating MI, the harsh environment of the ischemic heart, including inflammation and insufficient vascular supply, significantly reduces the cell retention and engraftment of MSCs, leading to a decreased therapeutic efficacy [29, 30]. It has been documented that only 0.44% of transplanted MSCs were observed in the heart at 4 days posttransplantation [31]. Improving MSC survival and engraftment posttransplantation in the infarcted heart to enhance the therapeutic efficacy is therefore vital. A growing body of studies has demonstrated that pretreatment via various strategies including hypoxia, growth factors, and pharmacologic agents to optimize MSCs for the hostile environment before transplantation robustly enhances their survival and engraftment after transplantation, promoting their therapeutic efficacy for MI [32, 33]. Pretreating MSCs with growth differentiation factor 11 was shown to improve cell survival and retention in the infarcted hearts of mice by promoting mitochondrial fusion, leading to better cardioprotective effects [34]. Recently, accumulating evidence reported that pretreatment of MSCs with adipokines including Asprosin and C1q/tumor necrosis factor-related protein-9 remarkably enhanced MSC survival in the ischemic microenvironment, thereby improving the therapeutic effects of MSCs in MI [35, 36]. Apelin, a novel adipokine, is involved in regulating many physiological processes including heart function, angiogenesis, and energy metabolism [36]. Whether pretreatment with Apelin-13 can improve MSC survival in ischemic heart tissue has yet to be examined. In the current study, we pretreated MSCs with Apelin-13 and transplanted them into the infarcted area of the heart in a mouse model of MI. The results revealed that preconditioning with Apelin-13 greatly improved the MSC survival in the ischemic heart tissue, leading to a superior therapeutic effect of MSCs in MI. Based on the current findings, we understand the possible mechanisms of Apelin-13 pretreated MSCs from two aspects: improved cell survival and enhanced paracrine functions. We showed Apelin-13 pretreatment improved MSC survival in the ischemic hearts compared to the nontreated MSCs (Figure 5). *In vitro* studies demonstrated that Apelin-13 pretreatment inhibited SD/H-induced MSC apoptosis by downregulating mitochondrial fission via the ERK pathway (Figure 2), indicating Apelin-13-MSC possessed superior ability to tolerate harsh environment. In addition, Apelin-13 pretreatment enhanced the paracrine proangiogenic properties of MSCs, as evidenced by elevated capillary and arteriole density *in vivo* (Figure 6), and increased tube formation and proangiogenic cytokine expression *in vitro* (Figure 3). However, a more detail information of Apelin-13 pretreatment on the MSC secretome, and the crosstalk between the transplanted Apelin-13-MSCs and the aborigines in the ischemic hearts, including cardiomyocytes, fibroblasts, and immune cells, needs further exploration.

Mitochondrial dynamics, mediated by fission and fusion, play a critical role in determining cellular death. Mitochondrial fission is mainly regulated by Drp1, which binds to the mitochondrial fission factor and localizes to the outer mitochondrial membrane, leading to mitochondrial fragmentation. Excessive mitochondrial fission causes mitochondrial ROS generation, leading to cell apoptosis [37]. Anoxia/reoxygenation treatment-induced miR-762 translocated into the mitochondria and caused mitochondrial fragmentation via targeting NADH dehydrogenase subunit 2, leading to cardiomyocyte apoptosis [38]. Cobalt chloride has been shown to trigger the apoptosis of human periodontal ligament stem cells by upregulating mitochondrial fission via activation of Drp1, effects that were partially abolished by Drp1 inhibitor mitochondrial division inhibitor-1 [39]. Consistently, our previous study showed that SD/H induced apoptosis of MSCs by activating mitochondrial fission [19]. In the current study, Apelin-13 pretreatment significantly reduced SD/H-induced MSC apoptosis by inhibiting mitochondrial fragmentation and ROS generation. Nonetheless, the underlying mechanisms remain unclear. Accumulating evidence has shown that the ERK signaling pathway plays an essential role in regulating mitochondrial fission via activating Drp1 phosphorylation [28, 40]. It has been reported that early ERK1/2 activation promotes Drp1-mediated mitochondrial fission and contributes to cell reprogramming [41]. In contrast, Yap overexpression attenuates septic cardiomyopathy by inhibiting mitochondrial fission via activation of the ERK pathway that in turn sustains cardiac function [42]. These contrary results suggest that the ERK signaling pathway mediates mitochondrial fission in a cell type and stimulus-dependent manner. In the current study, Apelin-13 treatment significantly downregulated SD/H-induced apoptosis of MSCs by inhibiting mitochondrial fission via activating the ERK signaling pathway, and these effects were partially abolished by the ERK inhibitor U0126. A growing body of evidence shows that the therapeutic efficacy of MSCs in MI is largely attributed to their paracrine effects, especially their angiogenic capacity. Interestingly, Apelin mediates cellular angiogenesis via multiple pathways [43, 44]. We also found that Apelin-13 pretreatment greatly enhanced the angiogenic capacity of MSCs, as evidenced by the increased capillary-like tube formation of HUVECs cultured with conditioned medium and enhanced capillary and arteriole density in the ischemic heart tissue. Our previous studies have documented that the ERK signaling pathway is involved in regulating the angiogenic capacity of MSCs [25, 45]. In this study, we also observed that inhibition of the ERK signaling pathway with U0126 significantly downregulated the angiogenic capacity of Apelin-13-treated MSCs, indicating that Apelin-13 stimulates the angiogenic capacity of MSCs by activating the ERK signaling pathway.

This study has some limitations that require further investigation. First, we proposed the activation of ERK signaling as one of the mechanisms involved in the Apelin-13 mediated effects. We focused on alteration in the ERK pathway based on the fact that Apelin-13 pretreatment inhibited SD/H-induced mitochondria fission (Figure 1), and that the ERK pathway is an important regulator in the process of mitochondria fission. However, different mechanisms other than the ERK signaling can be involved in the Apelin-13 mediated cytoprotection. Moreover, the situations might be complicated when MSCs are transplanted to the infarcted heart, and whether ERK signaling plays a role in the Apelin-13-MSC mediate effects *in vivo* needs further investigation. Second, the impact of endogenous Apelin -13 on MSC survival in the ischemic heart was not determined. Third, in the current study, human MSCs were transplanted into immunocompetent mice. Despite the low immunogenicity of MSCs, there is still a possibility of immune rejection that might affect the *in vivo* survival. We tracked very few MSCs in the diseased heart at 28 days post-MI; however, the long-term survival of Apelin-13-pretreated MSCs in the mouse heart following infarction needs further investigation. Fourth, in addition to improved cell survival and angiogenic capacity, whether Apelin-13 pretreatment can enhance the therapeutic effects of MSC-derived exosomes in MI remains to be addressed. Last but not least, we used bone marrow derived MSC (BM-MSC) in the current study. However, MSCs derived from primary sources and the conventional MSC manufacturing approaches are hampered by challenges with scalability and interdonor variability. Alternatively, MSCs derived from induced pluripotent stem cells (hiPSC-MSCs) are emerging as an attractive option [46, 47]. hiPSC-MSCs showed better proliferation, survival, immunomodulatory properties, and therapeutic efficacy for myocardial repair than BM-MSC and possessed the capacity to transfer mitochondria to rescue mitochondrial dysfunction [6, 48]. In accordance with their findings, our group showed that conditioned medium of hiPSC-MSC (iMSC-CdM) was superior to that derived from umbilical cord MSC in accelerating wound closure [45]. GMP-grade hiPSC-MSCs have been applied in acute steroid-resistant graft versus host disease (GVHD) in clinical trials [49]. hiPSC-MSCs might represent one of the future perspectives in MSC-based therapy.

## 5. Conclusion

Our study shows that pretreating MSCs with Apelin-13 greatly improves cell survival under SD/H challenge by downregulating mitochondrial fission via inhibition of the ERK signaling pathway and enhances cardiac function following infarction in mice. These results reveal a novel pharmacological approach to improve the cardioprotective efficacy of MSC-based treatment for cardiovascular diseases.

## Data Availability

The original contributions presented in the study are included in the article, and further inquiries can be directed to the corresponding authors.

## Supplementary Materials

Supplementary Materials Supplementary Figure 1: CCK-8 assay showed the cell viability of Apelin-13 treated MSCs under normoxic condition. Supplementary Figure 2: Brdu assay showed that MSC or Apelin-13-MSC did not induce proliferation of mice spleenocytes. Supplementary Figure 3: APJ expression in MSCs derived from different donors determined by qRT-PCR.
