# Supplementary material for: Apelin-13 Pretreatment Promotes the Cardioprotective Effect of Mesenchymal Stem Cells against Myocardial Infarction by Improving Their Survival
Source: Stem Cells Int. 2022 Mar 21;2022:3742678. doi: 10.1155/2022/3742678 (PMC8960019; doi:10.1155/2022/3742678)
Supplement: Supplementary Materials — Supplementary Figure 1: CCK-8 assay showed the cell viability of Apelin-13 treated MSCs under normoxic condition. Supplementary Figure 2: Brdu assay showed that MSC or Apelin-13-MSC did not induce proliferation of mice spleenocytes. Supplementary Figure 3: APJ expression in MSCs derived from different donors determined by qRT-PCR. [file 3742678.f1.docx]

**supplementary information**

**Apelin-13 pretreatment promotes the cardioprotective effect of mesenchymal stem cells against myocardial infarction by improving their survival**

Guona Chen^1,2#^, Xiaoting Liang^3#^, Qian Han^4^, Cong Mai^2^, Linli Shi^2^, Zhuang Shao^2^,

Yimei Hong^2^, Fang Lin^5^, Mimi Li^5^, Bei Hu^1^, Xin Li^2,1*^, Yuelin Zhang^2,1*^,

^1^School of Medicine, South China University of Technology, Guangzhou, China;

^2^Department of Emergency Medicine, Guangdong Provincial People's Hospital, Guangdong Academy of Medical Sciences, Guangzhou, China;

^3^Institute for Regenerative Medicine, Shanghai East Hospital, School of Life Sciences and Technology, Tongji University, Shanghai, China;

^4^Department of Respiratory Medicine, The First Affiliated Hospital of Guangzhou Medical University, Guangzhou Institute of Respiratory Health, State Key Laboratory of Respiratory Disease, Guangzhou, China;

^5^Research Center for Translational Medicine, Shanghai East Hospital, School of Medicine, Tongji University, Shanghai, China.

^#^These authors contributed equally to this study.

***Address correspondence to:**

Dr. Yuelin Zhang, MD, PhD or Dr. Xin Li, MD, PhD

Department of Emergency Medicine, Guangdong Provincial People's Hospital, Guangdong Academy of Medical Sciences, Guangzhou, China.

Tel.86-20-83827812-20974

E-mail: [zhangyuelin1999@163.com](mailto:zhangyuelin1999@163.com) (Yuelin Zhang); or [xlidoct@qq.com](mailto:xlidoct@qq.com) (Xin Li)

**Figure legend:**

**supplementary Figure 1.** CCK-8 assay showed the cell viability of Apelin-13 treated MSCs under normoxic condition.

supplementary Figure 1

**supplementary Figure 2.** Brdu assay showed that MSC or Apelin-13-MSC did not induce proliferation of mice spleenocytes.

supplementary Figure 2

**supplementary Figure 3. APJ expression in MSCs derived from different donors determined by qRT-PCR.**

supplementary Figure 3
